# Changes in ground beetle assemblages above and below the treeline of the Dolomites after almost 30 years (1980/2009)

**DOI:** 10.1002/ece3.927

**Published:** 2014-03-15

**Authors:** Roberto Pizzolotto, Mauro Gobbi, Pietro Brandmayr

**Affiliations:** 1 Dipartimento B.E.S.T., Università della CalabriaVia Bucci, I-87036, Arcavacata di Rende (Cosenza), Italy; 2 Sezione di Zoologia degli Invertebrati e Idrobiologia, Museo delle ScienzeVia Calepina 14, I-38122, Trento, Italy

**Keywords:** Carabids, dolomites, global change, mountain

## Abstract

Very little is known about the changes of ground beetle assemblages in the last few decades in the Alps, and different responses to climate change of animal populations living above and below the treeline have not been estimated yet. This study focuses on an altitudinal habitat sequence from subalpine spruce forest to alpine grassland in a low disturbance area of the southeastern Dolomites in Italy, the Paneveggio Regional Park. We compared the ground beetle (Carabidae) populations sampled in 1980 in six stands below and above the treeline (1650–2250 m a.s.l.) with those sampled in the same sites almost 30 years later (2008/9). Quantitative data (species richness and abundance) have been compared by means of several diversity indexes and with a new index, the Index of Rank-abundance Change (IRC). Our work shows that species richness and abundance have changed after almost 30 years as a consequence of local extinctions, uphill increment of abundance and uphill shift of distribution range. The overall species number dropped from 36 to 27, while in the sites above the treeline, species richness and abundance changed more than in the forest sites. Two microtherm characteristic species of the pioneer cushion grass mats, *Nebria germari* and *Trechus dolomitanus*, became extinct or showed strong abundance reduction. In Nardetum pastures, several hygrophilic species disappeared, and xerophilic zoophytophagous elements raised their population density. In forest ecosystems, the precipitation reduction caused deep soil texture and watering changes, driving a transformation from *Sphagnum*-rich (peaty) to humus-rich soil, and as a consequence, soil invertebrate biomass strongly increased and thermophilic carabids enriched the species structure. In three decades, Carabid assemblages changed consistently with the hypothesis that climate change is one of the main factors triggering natural environment modifications. Furthermore, the level of human disturbance could enhance the sensitivity of mountain ecosystems to climate change.

## Introduction

The study of qualitative and quantitative changes of animal assemblages is a key challenge in ecology. Such changes can be related to temporal (Gotelli et al. 2010) and climate (Parmesan and Yohe 2003) trends or triggered by occurrence or abundance changes of other species (White et al. 2006) and by long-term changes of abiotic factors (Dunson and Travis 1991). Our study refers to a qualitative and quantitative study, conducted in the Dolomites (southeastern Alps), aimed at performing a temporal comparison between the ground beetle assemblages sampled in the year 1980, in habitats above and below the treeline, versus those sampled almost 30 year later (2009) in the same sampling sites.

The Alps can be considered an ideal “experimental fields of nature” (Spehn and Körner 2010), and the Dolomites were selected as a key area because they are a unique study area to test the effect of climate change on insect assemblages, due to their relative low average elevation with respect to other mountain groups of the Italian Alps and their relative aridity conditions due to the high soil drainage. These particular features lessen the probability of finding cold habitats over large areas for keeping viable populations against the temperature increase. Moreover, the southeastern Dolomites are the only area of the Italian Alps where quantitative year samplings of several invertebrate taxa have been gathered in the past century with standard pitfall traps (Brandmayr 1988). In addition, the land use and management of the sampled area have undergone no remarkable changes in the past 30 years because all the sites are enclosed in the Paneveggio Regional Park which has been particularly targeted toward maintaining traditional forests and grasslands management.

Carabid beetles were selected as the key taxon because their ecology and biodiversity patterns are well known for the Alpine environments (Brandmayr et al. 2003), and it has been found that they clearly react to alpine climate changes (Gobbi et al. 2007, 2010, 2011; Brambilla and Gobbi 2013)).

The aim of our study is to quantify the changes that have occurred above and below the treeline in ground beetle assemblages structure after 30 years since the first survey. We tested whether: 1. species rank-abundance distribution (RAD) changed with time; 2. the populations above the treeline react differently with respect to those below; 3. there are single species that are more susceptible to climate change with respect to the whole assemblage. If climate change played the major role, we expect that the following: (1) microtherm species become extinct or show uphill shift as temperate species do; (2) hygrophilic taxa become less abundant; (3) there are species traits that enable some species to react accordingly to new climate conditions.

## Materials and Methods

### Study area

**Figure 1:**
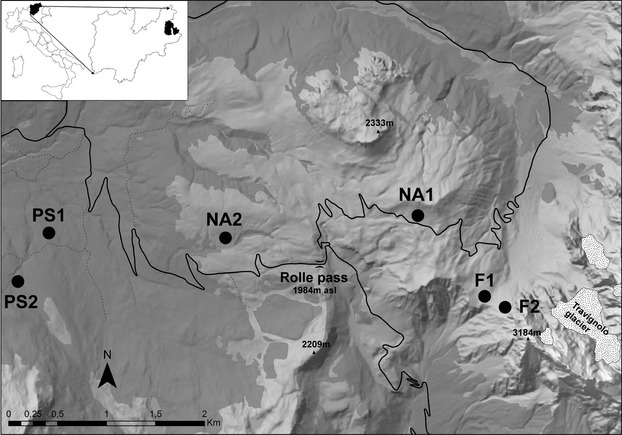
The study area is part of the “Paneveggio, Pale di S. Martino” Regional Park, in the SW Dolomites, around the Rolle Pass (46°17′48.15″N, 11°47′14.62″E). Upper left white area, at left Italy and in black, the Trentino Alto Adige region, at right, the Trento province and in black, the Paneveggio Park.

The study area (Fig. 1) is part of the “Paneveggio, Pale di S. Martino” Regional Park, in the SW Dolomites, around the Rolle Pass (1980 m a.s.l., 46°17′48.15″N, 11°47′14.62″E). It lies near the border between Trentino Alto Adige (inner Dolomites, Fassa, and Fiemme valleys) and Veneto (Piave river valley) regions. The bedrock is dolomite or ignimbrite, the vegetation is mainly conifer forests (spruce *Picea abies*, larch *Larix decidua*, mountain pine *Pinus mugo*) up to 1800 m, and alpine grass mats above the treeline, joined by a wide strip of zoo-anthropogenous hay-meadows and pastures, nearby always with dense moor mat grass covering (Nardetum alpigenum), due to the high cattle grazing pressure. The Nardetum stands were grazed in 1980 as well as in 2008–2009, while the alpine grass mats were not grazed in either time. On the whole, at each time two forests, pastures and alpine prairies were sampled.

The climate of the study area and its changes in the last few decades have been studied on the basis of the data available from the climatic database of the Trento province (http://hydstraweb.provincia.tn.it/web.htm). Long time-series data are available for only three meteorological stations around the study area; therefore to obtain a reliable picture of the climate change of the study area, the multidecadal oscillation of mean temperature and mean of the total rainfall of the Predazzo town (1005 m a.s.l., data from 1926 to 2005), S. Martino town (1450 m a.s.l., 1925–2007), and Rolle pass (2012 m a.s.l., 1925-present) meteorological stations have been analyzed. Mean temperature among the three stations (common data available up to 2005) was computed for drawing the 10-year moving average series linked to the study area, and then, the series was compared with the CRUTEM4 series (Jones et al. 2012), which is a dataset of global historical near-surface air temperature anomalies, showing that the global temperature trend is consistent with the global warming hypothesis. The correlation of study area mean temperature with the CRUTEM4 and along the studied time frame (i.e., the temporal trend within 1980–2008) was evaluated, while only the last was evaluated for rainfall data.

### Sampled sites

Carabids were sampled in six sites in 1980 (Brandmayr and Zetto Brandmayr 1988), and then, the same sites were resampled in 2008 or 2009, that is, after a period of about 30 years. The exact positions of the pitfall traps were refound by historic photographs, maps drawn in 1980 and on the field by P. Brandmayr. Sampling site characteristics (ordered by increasing altitude) are as follows: (see Table 1 for the main topographic features, complete phytosociologic inventories are reported in Boiti and Boiti Saffaro (1988)).

**Table 1:** Main features of the sample sites, ordered by increasing altitude.

Sample site	Altitude m a.s.l.	Aspect	Slope°	Veget %
PS1 (PS1_08) Homogyno-Piceetum sphagnetosum	1650	NW	10	80–90
PS2 (PS2_08) Homogyno-Piceetum myrtilletosum	1780	NNE	25	60–90
NA2 (NA2_09) Nardetum alpigenum	1910	S	15	100
NA1 (NA1_09) Nardetum alpigenum	2170	SSW	20	100
F1 (F1_08) Caricetum firmae	2200	NNW	35	60
F2 (F2_08) Caricetum firmae (colonist)	2250	NW	35	20

The suffix _08 means the same site resampled in 2008, while _09 is for 2009.

PS1 (PS1_08, the same site resampled in 2008). Homogyno-Piceetum sphagnetosum on raw humus soil on ignimbrite moraine bedrock. Subalpine spruce forest with dense blueberry (*Vaccinium*) and *Sphagnum* moss understory.

#### PS2 (PS2_08)

Homogyno-Piceetum myrtilletosum on ferric podsol on ignimbrite moraine bedrock. Spruce forest *Sphagnum* poor, lighten facies are invaded by tall forbs of the Adenostyletalia. Some years before 2008, the site was clear cut, so we had to shift to a new site to the nearest similar forested area (a 100 m away). Sometimes moderate grazing has been recorded.

#### NA 2 (NA2_09)

Nardetum alpigenum on clay rich podsol on “Val Gardena” sandstone. Mat grass pasture probably derived from a pristine *Larix* and *Pinus cembra* forest, on a humid soil. The site is patched with wet areas and every year severely trampled by cows.

#### NA1 (NA1_09)

Nardetum alpigenum on well-developed podsol (with humoferric depots) on fine-grained Werfen sandstone. Mat grass pasture at the limit of the treeline possibly derived from a pristine *Rhododendron* dwarf shrub environment. Grazing pressure lower than in NA2.

#### F1 (F1_08)

Caricetum firmae on rendzina soil. *Carex firma* calcareous grassland on stabilized dolomitic debris.

#### F2 (F2_08)

Caricetum firmae on protorendzina soil on mobile clastic debris. Pioneer calcareous grassland on unstable dolomite rock scree.

In each site, a row of six pitfall traps, spaced 10 m apart, was used, and the catches of the traps were pooled in order to optimize the catch and to overcome occasional trap losses (Kotze et al. 2011). Carabids were trapped continuously for an entire season of carabid activity, that is, between June and September (temperature being the limiting factor), and the traps were emptied every 20–30 days; thus, approximately three trap collections were made up as 1-year sample. The traps were plastic cups of 9 cm at the upper diameter and 11 cm deep, a small hole near the border prevented overflowing by rain. They were filled each time with 200 cl of a standard mixture of wine vinegar and 5% formalin. The sampling techniques remained the same in terms of trap type, trap location, attractive solution and sampling period.

### Diversity analysis

Data have been analyzed in R (R Core Team 2013) by means of the packages vegan (Oksanen et al. 2013), cluster (Maechler et al. 2013), rich (Rossi 2011), Rcmdr (Fox 2005).

The before vs. now overlapping of the species assemblages (i.e., community level) has been analyzed by means of cluster analysis (Maechler et al. 2013), on the basis of chord distance dissimilarity and minimum variance clustering method (see Pielou 1984).

The variation in the species richness (i.e., number of species) and abundance has been evaluated between 1980 and 2008–2009, and the abundance was computed as annual activity density (aAD), that is, mean number of individuals per trap in the standard period of 10 trapping days. Bootstrapping (Rossi et al. 2006; Rossi 2011) was used to estimate the species richness. The technique produces a large data set (i.e., *n* samples, where *n* is under subjective decision) consistent with the observed data, by randomly sampling with replaced variables from the actual data set, giving *n* new samples as if we get them from *n* sites similar to the one actually sampled. Such a technique allows for the computation of confidence intervals that are meaningful for the actual data.

Bootstrapping was applied (“rich” function in Rossi 2011) to 1980 and to 2008–2009 data sets for comparing the actual number of sampled species with the expected (bootstrapped) number for evaluating the eventual amount of data lacking. The expected number of species has been evaluated on the basis of the probability of encountering the less frequent species, that is, the expected number is the theoretical number of species that one can encounter if even the less catchable species would have been caught in all the sites (Smith and van Belle 1984; Colwell and Coddington 1994; Rossi 2011).

The species richness of the two data sets has been compared on the basis of their rarefaction curves (i.e., species richness against bootstrapped sampling intensity) after rescaling the dataset with the largest individual density, as outlined in Rossi (2011).

The minimum percentage similarity (i.e., Renkonen index) of “before versus now” pair of sites has been computed for describing the species richness and abundance change over time. The index is useful when comparing absolute abundances from different type of environments (Pontasch et al. 1989).

The variation in the structure of the species assemblages has been evaluated on the basis of rank versus abundance species plots (Waite 2000; Magurran 2004), that is, by comparing the shape of the actual RAD with theoretical models of species abundance distribution (SAD) for each site in the past and now. Several models are often used for summarizing community data sets because they provide more comprehensive description of the data. Among the most used, the broken-stick model is proposed as null model by Oksanen et al. (2013), and the niche pre-emption model is consistent with low diversity and few abundant species, that is, low equitability (Magurran 2004), while when the species are more equitably distributed, the lognormal and the Mandelbrot models fit well (Sughiara 1980; Magurran 2004). The SAD best describing each species assemblage was selected on the basis of the Akaike information criterion (Oksanen et al. 2013).

Changes in species structure composition have been quantified by performing a new ad hoc index, in the attempt to measure the RAD variation in “before versus now” pair of sites.

For a given set of species, if two samples (e.g., *A* and *B*) taken from that set, we find all the species of the set with the same rank order and then *A* and *B* show the same species distribution, that is, there is no RAD difference between *A* and *B*. If one species (e.g., sp1) occupies different position in the RAD of the samples, then the RAD difference between *A* and *B* is equal to the rank order shift of sp1 between *A* and *B*. If more than one species occupy different position, then the index is given by the sum of all species shifts. This means calculating city-block distance between *A* and *B*, where each species is weighted on the basis of its rank order. The distance is then divided by the number of the species in that set to make the index comparable among the sites.

The formula may be expressed as follows 

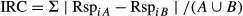


We call this index IRC: Index of Rank-abundance Change, and in our case, *A* is the site sampled in the past, and B is the same site sampled in the present, Rsp_*i*_ is the rank order of the *i*th species, with *i* ranging from 1 to *A* U *B* (i.e., the number of species from the union of *A* with *B*).

It will be helpful for the discussion to evaluate the importance of two well-known carabid species traits, that is, hind wings morphology, where fully winged (macropterous) species are typical of unstable or disturbed (i.e., human affected) habitats (den Boer et al. 1980; Brandmayr 1983, 1991; Rainio and Niemelä 2003; Pizzolotto 2009) and food preference, where zoophytophagous species follow xeric vegetation (i.e., climate affected).

## Results

**Figure 2:**
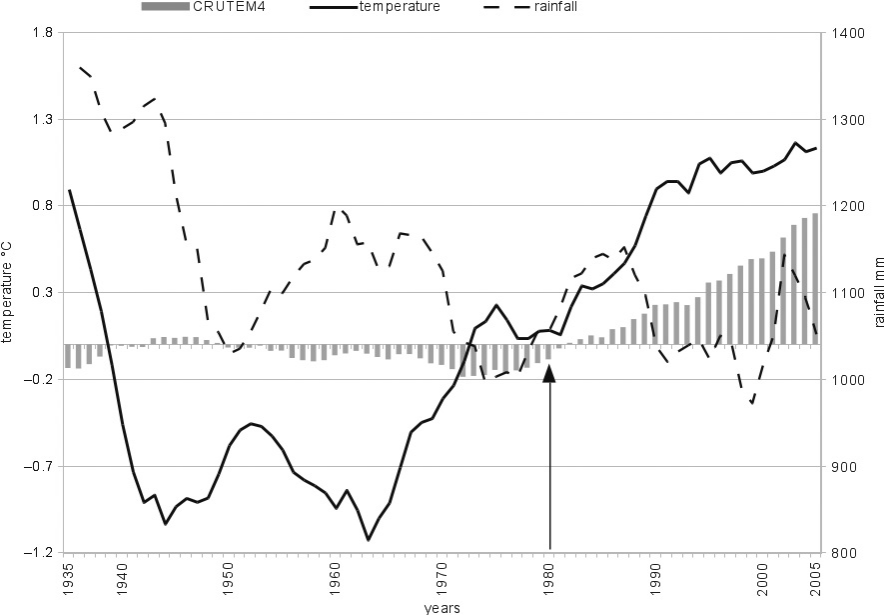
Ten-year moving average of mean temperature and mean of the total rainfall in three stations in the study area. Long-term data (1926–2005) are available for only Predazzo (1005 m a.s.l.), S. Martino (1450 m a.s.l.), and Rolle pass (2012 m a.s.l.).

On the basis of the data available in the Trento province database (http://hydstraweb.provincia.tn.it/web.htm), the climate of the Rolle Pass and of the surrounding Dolomite peaks has been classified as temperate–humid type VI by Walter and Lieth (1960). In Fig. 2, the 10-year moving average of mean temperature and mean of the total rainfall averaged out among three meteorological stations around the study area were drawn superimposed upon temperature anomalies (i.e., CRUTEM4 dataset). Temperature shows significative high-positive correlation with CRUTEM4 both for the whole available time series (i.e., 1936–2005, *r* = 0.7, *P* < 0.0001) and for the time frame of the study (i.e., 1980–2005, *r* = 0.9, *P* < 0.0001); furthermore, it shows significative positive increase with time both along the whole available time series (*r* = 0.8, *P* < 0.0001) and along the time frame of the study (*r* = 0.9, *P* < 0.0001. See also supplementary Figs. S1 and S2).

Such correlations are not as strong for the mean total rainfall, which decreased with time both along the whole available time series (*r* = 0.66, *P* < 0.0001) and along the time frame of the study (*r* = 0.39, *P* = 0.05). The rainfall dropped from 1360 mm in 1936 to 1053 mm in 2005; moreover, it is noteworthy that in the time frame of the study, temperature and rainfall were negatively correlated (*r* = 0.5, *P* = 0.006. See also supplementary Figs. S1 and S2).

**Figure 3:**
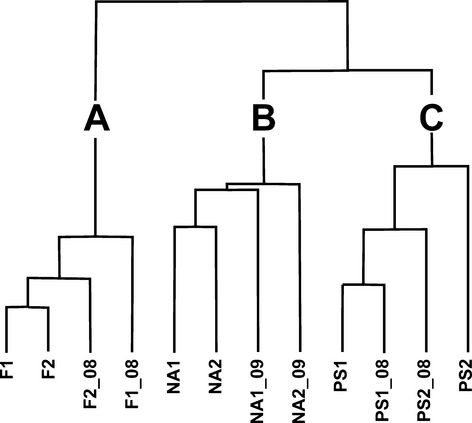
Classification of the sample sites on the basis of chord distance dissimilarity and minimum variance clustering method. A, grasslands; B, pastures; C, woods. Each pair of before–now samples falls in the same group, thus showing no intergroups similarity variation after almost 30 years.

The classification of the data is depicted in the dendrogram of Fig. 3, where three groups of sites are detectable. The evidence argues for the absence of between-groups similarity variation during the almost 30 years considered in our research, because each pair of before–now samples falls in the same group. The within-groups similarity change is evident for grasslands and pastures, because 1980 samples are clustered separately from the 2008–2009 samples; that is, F1 and F2 are more similar to each other (Fig. 3 group A) as compared with F1_08 and F2_08, respectively, and the same holds true for the pastures (Fig. 3 group B). Such a variation is not evident for the forest samples (Fig. 3 group C).

From the six pairs of sampled sites, a total of 36 species have been collected, 32 of which in 1980, accounting for aAD of about 29 specimens per trap, while 27 species have been collected in 2008–2009, accounting for aAD of about 59 specimens per trap. Species richness reduced, while species abundance increased over the studied time interval (see Table S1).

Twenty-three species were sampled in 1980 and 2008–2009, while nine species in 1980 only and 4 in 2008–2009 only, leading to a turnover that shrinked the fauna sampled in 2008–2009.

The expected species number in 1980 is 37 (c.i. 29–46), while the species sampled are 86% of the expected ones. In 2008–2009, the expected species number is 31 (c.i. 25–37), while the species sampled are 87% of the expected ones. The actual species richness is within confidence intervals for both sampling campaigns; hence, sampling intensities were adequate in the studied sites.

**Figure 4:**
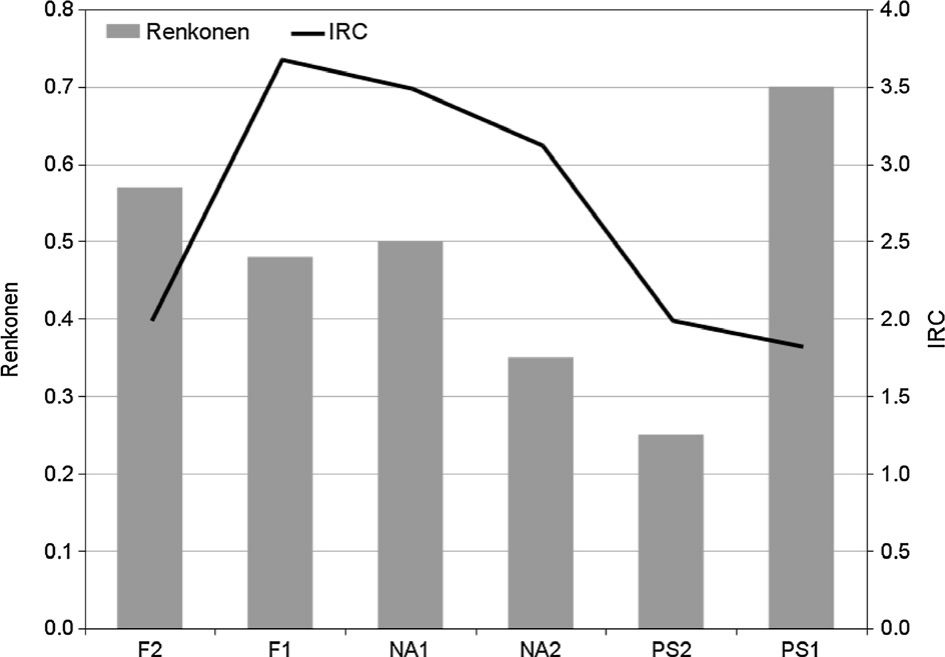
Diversity indexes. Renk., Renkonen minimum percentage similarity versus IRC, Index of Rank-abundance Change.

We then calculated the Renkonen index of “before versus now” pair of sites to evaluate changes in species richness and abundance over the time under consideration. The index evaluated in nonforest ecosystems shows the lowest similarity (0.35) for the NA2 pair of samplings (Fig. 4), which is a pasture reclaimed from swiss pine–larch vegetation, while the pasture at the higher altitude (NA1) shows intermediate values of similarity (0.50). Apparently, the same holds for the high-altitude prairies, where F1 shows low similarity (0.48) compared with F2 (0.57), that is, at higher altitude. In forest ecosystems (Fig. 4), the percentage similarity after 30 years remains high (about 0.70) in the core of the forest, but the upper forest border shows deep changes of the community (less than 0.30). The IRC index behaves in an opposite way with respect to the Renkonen index (Fig. 4): the highest changes are recorded for the mat grass pastures, but F1 shows a much higher species turnover, while in the forests, the changes at the upper border are more evident.

**Figure 5:**
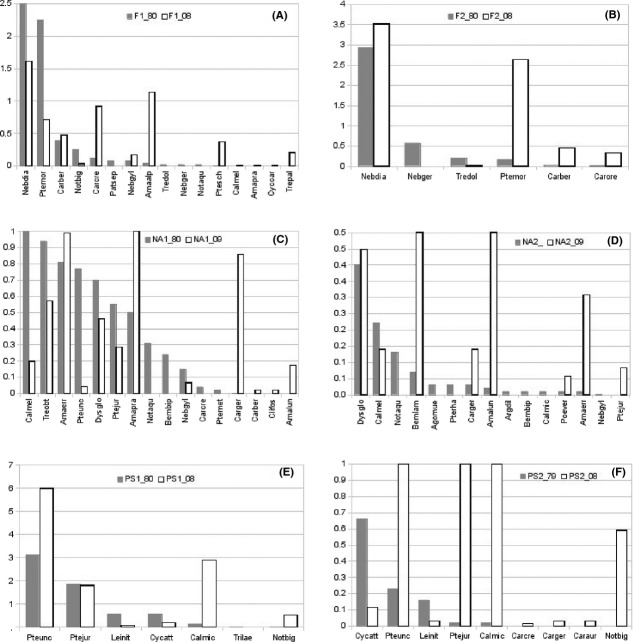
(A–F). Temporal comparison (1980–2008) between the species assemblages composition in the sampled sites.

The comparison of the observed species RADs with the models (i.e., SADs) shows that in the past, the nonforest ecosystems were more similar to the mandelbrot or broken-stick SAD model, while now, they are better depicted by the curve following the niche pre-emption SAD model. No changes are evident for forest sample sites (Table 2). The evaluation of species rank reordering shows that after 30 years, the nonforest ecosystems are the ones most affected by species abundance changes (Fig. 5E, F).

**Table 2:** Species abundance distribution best fitted in 1980 versus 2008–2009.

Site	1980	2008–2009
F2	Mandelbrot	Pre-emption
F1	Lognormal	Pre-emption
NA1	Broken stick	Pre-emption
NA2	Mandelbrot	Pre-emption
PS2	Pre-emption	Pre-emption
PS1	Lognormal	Pre-emption

The temporal comparison (1980–2008) between the species assemblages composition in the alpine prairies (sites F1 and F2) shows that the most remarkable variation is the disappearance of *Nebria germari* and the strong abundance reduction in *Trechus dolomitanus*. In the F1 site, the number of species remained the same, but (see Fig. 5A) four microtherm or hygrophilic species disappeared (*Patrobus septentrionis*,*Notiophilus biguttatus*,*T. dolomitanus*, and *N. germari*), and were replaced by new four species (*Cychrus caraboides*,*T. pallidulus*,*Amara praetermissa*, and *Calathus melanocephalus*). The alpine prairie F2 shows a decrement in species richness from six to five, with the loss of *N. germari*. Species abundance (aAD) in the prairies F1 and F2 shows an increment for *Carabus bertolinii*,*C. creutzeri*,*Amara alpestris*, while *N. diaphana* and *Pterostichus morio* decrease in F1, but increase in F2 (see Fig. 5A–B).

The temporal (1980–2009) comparison between the species composition in the pastures NA1 and NA2 shows the following changes: in NA1, four species disappeared (*N. aquaticus, Bembidion bipunctatum, C. creutzeri,* and *P. burmeisteri*), while three new species appeared (*C. bertolinii, Clivina fossor*, and *Amara lunicollis*) and *C. germari* increased markedly its population density (Fig. 5C). On the other hand, NA2 lost seven hygrophilic species (*N*. *aquaticus, A. mülleri, P. rhaeticus, B. bipunctatum, C. micropterus, A. diligens, and N. gyllenhali*) and a new one appeared in 2009 (*P. jurinei*). Worth of notice is the abundance decrement of *Calathus melanocephalus* in NA1, which is a consequence of lower grazing pressure, while *C. germari* increase in NA1 and NA2 (see Fig. 5C, D). In both mat grass stands, the abundance of *Amara* species is increasing, most species belonging to this genus are xerophilic seed feeders.

The forest sites showed contrasting variations. In the PS1 spruce stand, one species was lost (*Trichotichnus laevicollis*), while a strong variation appeared in PS2 with three new *Carabus* species (*C. creutzeri*,*C. auronitens,* and *C. germari*). Both PS1 and PS2 show a strong increment of individuals in *P. unctulatus*,*C. micropterus,* and *P. jurinei* (see Fig. 5E, F).

Interestingly, the species diversity variation documented in Fig 5, and Table S1 shows an uphill shift of some species ranges, leading them to become part of species groups of which they were not in the past.

## Discussion

The results show that almost 30 years ago, the carabid populations of the Dolomites were different both for the environmental and altitudinal distribution and that their assemblages show deep changes in species/abundance structure.

The intragroup diversity outlined in the classification gives evidence for an uphill shift affecting the distribution range of some species, rather than of the communities, thus introducing strong modifications in the SADs of the sampled sites.

The model best describing the RAD of the species assemblages shows that in high-altitude ecosystems, RAD changed from models (mandelbrot, lognormal, broken stick), where a slow successional process is presumable (Magurran 2004) because new colonists are ecologically more demanding (Sugihara 1980; Brambilla and Gobbi 2013), to the niche pre-emption model, where low diversity communities are dominated by a limited number of abundant species (Waite 2000), with consequent decrease in species equitability. Such a variation shows a regression toward the first stages of the ecological succession, with the consequent reduction in the species assemblage complexity.

Even if the expected number of species and SAD likelihood similarity are computed without weighting any quality of the species themselves (e.g., dispersal power, diet, stenotopy), it is worth noticing that the number of figures above shows a consistent trend in species and information loss. Furthermore, the higher species abundance in 2008–2009 makes the less catchable species more easy to be intercepted; therefore, if a rarefaction curve is computed on the basis of the rescaled number of individuals, as if they showed the same abundance as in 1980, then the expected number of species for 2008–2009 would be 19, that is, a value near to half of 32.

### Changes above the treeline

Our results show that the faunal composition changed in high-altitude ecosystems, as a consequence of local extinction, uphill increment of abundance and uphill shift of distribution range. The snow duration and the temperature gradients are known to be the main factors affecting distribution and abundance of carabids above the treeline (Mani 1962; Kühnelt 1968; Sota 1994, 1996). On the basis of this assumption, we can assert that the changes observed in the species distribution could be linked to the climate changes.

This is particularly true at least for the following species. *Nebria germari*, which has been sampled by Brandmayr and Zetto Brandmayr (1988) in poor developed, mostly stony and sparse vegetation soils (pioneer cushions), is usually present above the alpine prairies, in soils with scarce vegetation (<35%), scarce organic matter (<5%), pronounced interstitial cavities (Kaufmann and Juen 2002), and beneath perennial snows, that is, between 2400 m and 3000 m in the Dolomites. *Trechus dolomitanus* is an endemic species, tied to the environment near the snow belt. Both species can be found in the alpine prairies in North facing slopes and stony soils and were considered guide species of the pioneer vegetation belt of screes in the Dolomites (Brandmayr and Zetto Brandmayr 1988). Given the sharp environmental adaptation of these species, it is reasonable to consider the temperature increase as the main factor responsible for their local extinction/strong reduction.

The uphill abundance raise of *C. bertolinii* and *C. creutzeri* can be explained as temperature driven, because even if these species show a peak in, or are tied to, alpine prairies, it has been found (Brandmayr and Zetto Brandmayr 1988; Brandmayr and Pizzolotto 1989) that they can tolerate poor developed soils if the temperature is not an obstacle to their biologic cycle. Similarly, the seed feeder *Amara alpestris* shows an increment in F1, where it was not so abundant in the past, probably as a consequence of soil drying and increased availability of gramineous plant seeds.

The uphill shift of *N. diaphana*, which shows a remarkable increment in F2 and decrement in F1 in the 1980–2008 period, is probably the consequence of soil drying and of the temperature gradient change in these last 30 years, which forced this species to retreat to more favorable environments, because the limiting factors of its biologic cycle are humidity and low soil temperature (Brandmayr and Zetto Brandmayr 1988). *Nebria diaphana* differs from *N. germari* in its ability to colonize deep soil crevices and the subterranean environment (Brandmayr and Zetto Brandmayr 1988), whereas the second species is strictly bound to cool soil surfaces with scree, boulders and gravel and snow beds or near glacier snouts (Kaufmann and Juen 2002; Gereben-Krenn et al. 2011).

The pastures show remarkable variations for three species at least. *Carabus germari*, a thermophilic species widespread in many environments (Turin et al. 2003), was recorded in the past century only in pastures below 2000 m, while in 2009, it was abundant above 2100 m (NA1), where temperature increment and grazing decrement probably play a synergic positive role for the distribution of this species. The abundance dropping of *Calathus melanocephalus* is probably due more to the grazing decrement than to climatic change, because this species is tied to heavy soil structure. Worth of notice is the local extinction of *N. aquaticus* in 2009, because this is a hygrophilic species tied to cooler soils.

Concerning the overall species assemblage characteristics, it is helpful for the discussion to parallel the IRC index with two well-known carabid species traits, that is, hind wings morphology, where fully winged (macropterous) species are typical of unstable or disturbed (i.e., human affected) habitats (den Boer et al. 1980; Brandmayr 1983, 1991; Rainio and Niemelä 2003), and food preference, where zoophytophagous species follow xeric vegetation (i.e., climate affected).

**Figure 6:**
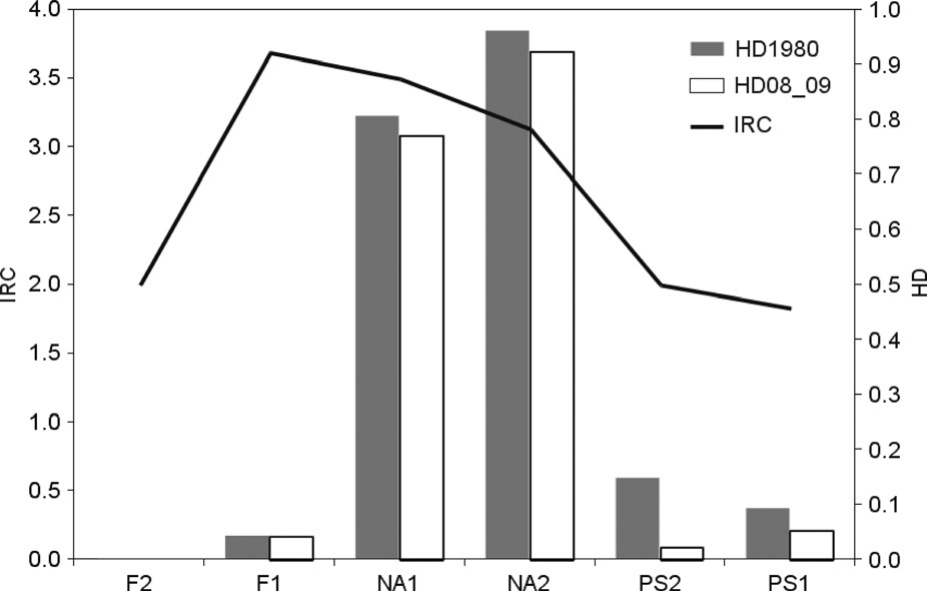
HD = species with high dispersal power, expressed as percentage of activity density (AD) of macropterous and dimorphic on the total annual activity density, plotted on IRC, Index of Rank-abundance Change.

**Figure 7:**
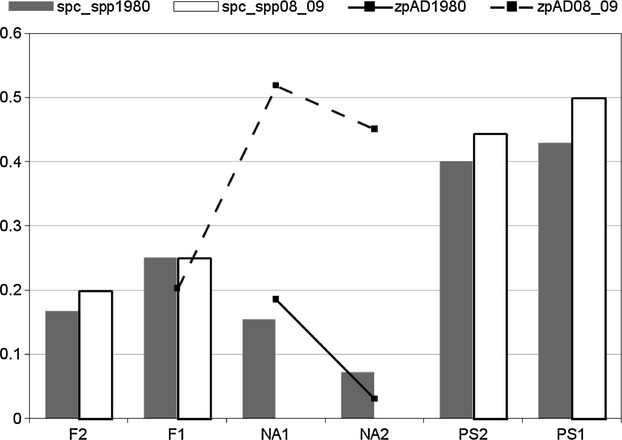
Incidence of specialized predators (spc_spp.,% of species) versus opportunistic zoophytophagous individuals (zpAD,% of AD).

It is remarkable that the IRC index is high in disturbed habitats (see Fig. 6), especially in the Nardetum stands, where grazing pressure is at the peak, and where the abundance of macropterous species is high. At the same time, specialized predators drop to zero in these pastures (Fig. 7), while xerophilic zoophytophagous species (genus *Amara*) “jump” to a new maximum. Such figures show that grazing disturbance plays a synergic role with climate changes in affecting carabid distribution.

### Changes under the treeline

Our results show that the faunal composition changed also in forest ecosystems.

The presence of *C. auronitens* in forest ecosystems around 2000 m was never recorded before 2008, when it has been sampled in PS2 and in a spruce sample site not included in pre-existing data. This species is a predator strictly linked to forest ecosystems, where it is able to climb on tree trunks for feeding (Turin et al. 2003). A constant mild warm climate during spring is the limiting factor for the settling of permanent populations (Turin et al. 2003), whose nocturnal activity is not easily intercepted by pitfall traps. Given such a particular adaptation to forest environments, and the warm spring limiting factor, the gradual temperature increasing in the last three decades is clearly the most important factor for the viability of the populations above 2000 m.

Similarly, the synergy between treeline uphill expansion and climate warming could be the most important factor explaining the abundance increase in *Calathus micropterus*,*P. jurinei*,*Pt. unctulatus,* and *N. biguttatus* in forest ecosystems. The first three species lived in the past century in suboptimal conditions, because the soil of the Paneveggio forest was peaty and covered by a thick *Sphagnum* layer, which restricted their larval activity to the soil surface. These generalist predators find now in the deep humus layers a rich prey supply and better opportunities for larval space use. On the contrary, we recorded an abundance decrement of *Leistus nitidus* as a consequence of the soil humidity decrease, visually detectable by the astonishing reduction in the *Sphagnum* covering with respect to the past, furthermore supporting the climate causality. The IRC index and Renkonen similarity argue for a very stable forest core (PS1) and a much less stable forest border (PS2); more exposed to warm-preferring intruders coming from the neighboring open lands, the species structure remains dominated by brachypterous forms and specialized predator species, but soil invertebrate activity increased by a factor of 10 (see Figs. 4, 6 and 7).

Our data demonstrate that the carabid species assemblages experiencing the highest variation are those located above the treeline, where population extinctions crowded. Two main variations have been revealed in the species assemblages: 1. changes on the species spatial distribution and 2. changes on species abundance (aAD). The former is in agreement with the ecological knowledge on the fauna reaction to climate warming (Beever et al. 2011), while the latter opens a new key point on the interpretation of the climate change effects on the animal communities. Looking at the data summarized in Table 3, it seems that at least some species traits, cold preference, hygrophily, and food preferences are responsible for ground beetle assemblage changes that reflect climate warming and precipitation decrease, whereas the unchanged species' dispersal power (see Fig. 6) indicates the more or less unchanged disturbance level of the sites. The overall faunistic balance suggests that the area is characterized by a process typical of the interglacial periods (Holdaus 1954; Drees et al. 2010), where alpine environments provide shelter for pleistocenic endemic species, while the uphill shift of forest ecosystems provides the corridor for the dispersion of thermophilic species, but quantitative data show that such a process has undergone an acceleration in the last 30 years. This study highlights that carabids proved to be reliable early warnings when they respond to the fast retreat of the ice-affected area as in the case of *N. germari*; furthermore, they play an important role as “day-after” warnings when they mirror slow vegetation changes as the case of *C. auronitens* showed.

**Table 3:** Synthesis of species number and abundance changes after almost 30 years.

Sample site	F2 – 2250 Caricetum firmae	F1 – 2200 Caricetum firmae	NA1 – 2170 Nardetum	NA2 – 1910 Nardetum	PS2 – 1780 Piceetum	PS1 – 1650 Piceetum
Number of spp.	5 (−1)	12 (–)	12 (−1)	8 (−6)	9 (+4)	6 (−1)
Microtherm spp.	1 extinct 1:−85%	3 n.r.				
Hygrophilic spp.			4 n.r.	7 n.r.	1:−80%	1:−85%
Thermophilic spp.		3 new spp.	2 new spp. 1 sharp i.	2 sharp i.	3 new spp. 1 sharp i.	
Specialized zoophagous (AD %)	+4%	–	−15%	−7%	+4%	+7%
Zoophytophagous (seed feeders AD %)	–	+20%	+32%	+42%	–	−0.01%

Extinct means a dense population disappeared; n.r. means not recorded and refers to sparse populations, with values of aAD >0.50 in 1980; sharp i. means sharp increase, that is, low or 0 aAD to >0.50; the symbol “–” means no change.

## Supporting Information

Figure S1 Pearson 1936–2005.

Figure S2 Pearson 1980–2005.

Table S1 Species annual Activity Density in the sample sites.
