# Supplementary figures and images for: Changes in ground beetle assemblages above and below the treeline of the Dolomites after almost 30 years (1980/2009)
Source: Ecol Evol. 2014 Mar 15;4(8):1284–94. doi: 10.1002/ece3.927 (PMC4020689; doi:10.1002/ece3.927)

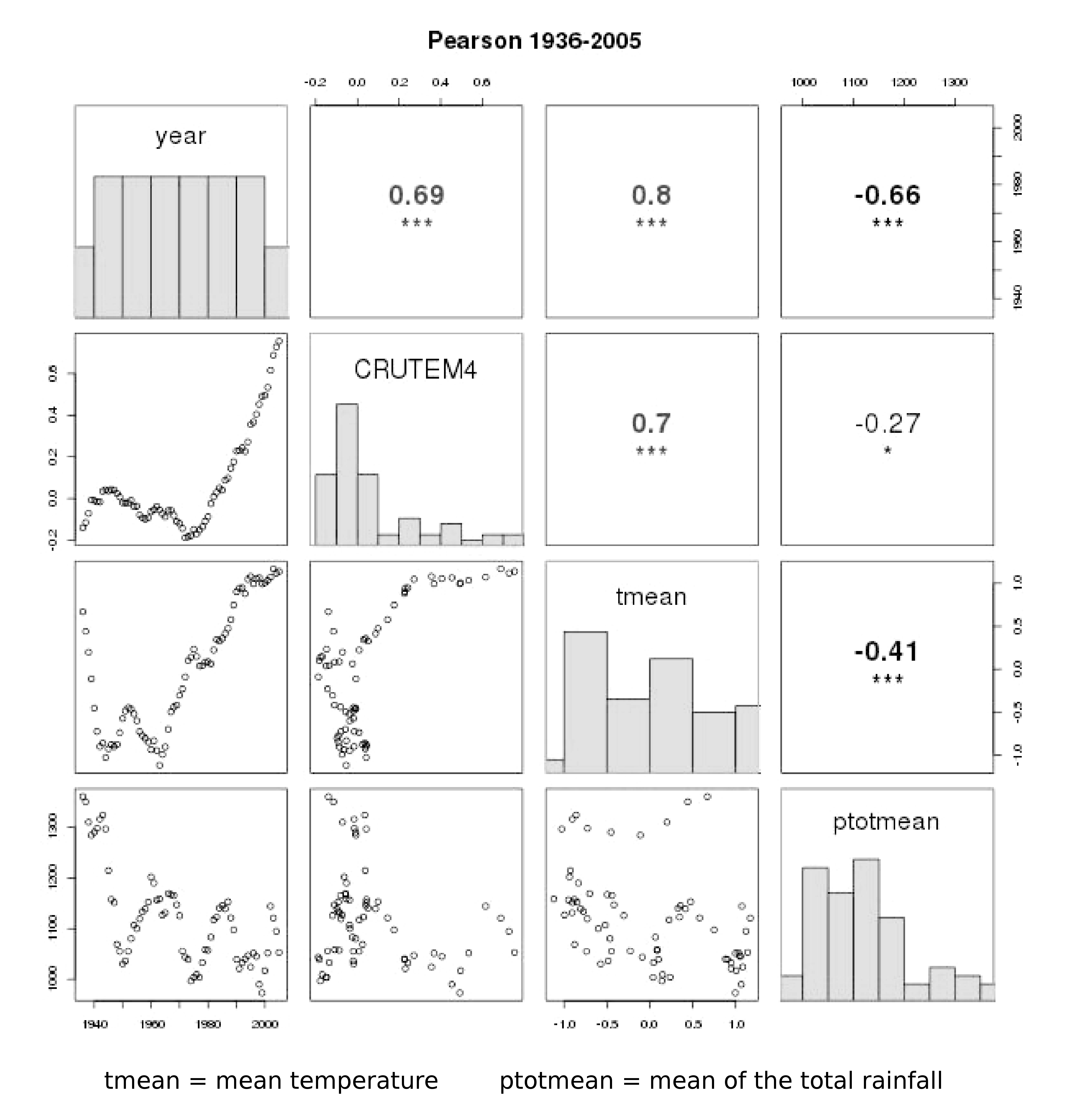

Supplement: Figure S1 — Pearson 1936–2005. [file ece30004-1284-sd1.jpg]

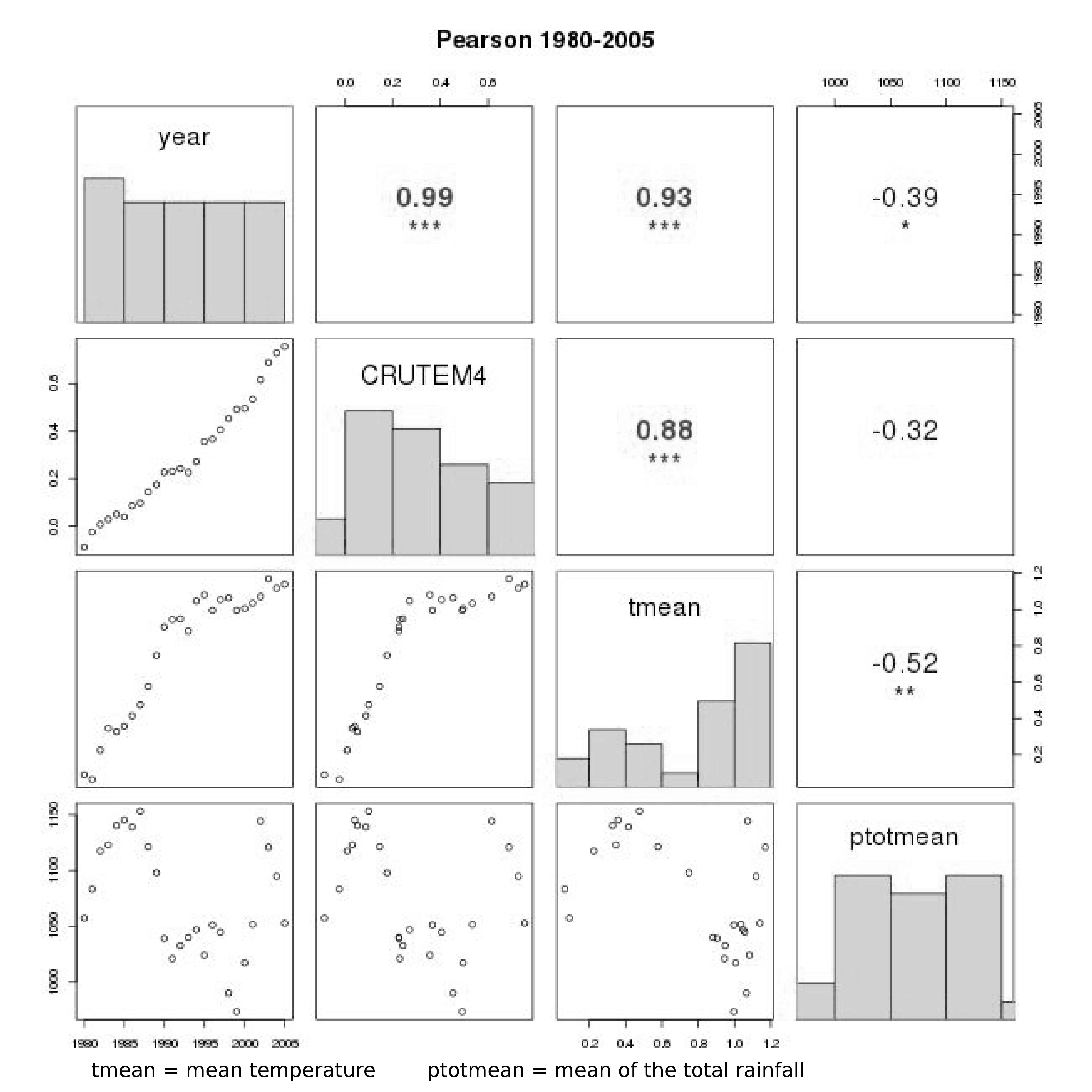

Supplement: Figure S2 — Pearson 1980–2005. [file ece30004-1284-sd2.jpg]
